# Fabrication of Polyethyleneimine-Functionalized Magnetic Cellulose Nanocrystals for the Adsorption of Diclofenac Sodium from Aqueous Solutions

**DOI:** 10.3390/polym14040720

**Published:** 2022-02-13

**Authors:** Xiaoyan Zhu, Jiaqi Tong, Hangzhen Lan, Daodong Pan

**Affiliations:** 1 State Key Laboratory for Managing Biotic and Chemical Threats to the Quality and Safety of Agro-Products, Ningbo University, Ningbo 315211, China; zhuxy@nbyjg.com (X.Z.); lanhangzhen@nbu.edu.cn (H.L.); 2 State Key Laboratory of Food Contact Material Testing, Ningbo Academy of Inspection and Quarantine, Ningbo 315048, China; tyrles@yeah.net; 3 College of Food and Pharmaceutical Sciences, Ningbo University, Ningbo 315211, China

**Keywords:** diclofenac sodium, magnetic nanocrystalline cellulose, polyethyleneimine, adsorption

## Abstract

Diclofenac sodium (DS), one of the most used non-steroidal anti-inflammatory drugs worldwide, is often detected in wastewater and natural water. This drug is ecotoxic, even at low concentrations. Therefore, it is essential to fabricate low-cost adsorbents that can easily and effectively remove DS from contaminated water bodies. In this study, a polyethyleneimine (PEI)-modified magnetic cellulose nanocrystal (MCNC) was prepared with a silane coupling agent as a bridge. TEM, FTIR, XRD, and VSM were used to demonstrate the successful preparation of MCNC-PEI. This composite adsorbent exhibited efficient DS removal. Furthermore, the adsorption performance of MCNC-PEI on DS was optimal under mildly acidic conditions (pH = 4.5). Adsorption kinetics showed that the adsorption process involves mainly electrostatic interactions. Moreover, the maximum adsorption capacity reached 299.93 mg/g at 25 °C, and the adsorption capacity only decreased by 9.9% after being reused five times. Considering its low cost, low toxicity, and high DS removal capacity, MCNC-PEI could be a promising adsorbent for treating DS-contaminated water.

## 1. Introduction

Diclofenac sodium (DS) is a popular non-steroidal anti-inflammatory drug and is often used in clinical treatment of rheumatic diseases. Worldwide consumption of DS is approximately 1443 tons [1]. Owing to the huge production and usage of DS globally, it is frequently detected in surface water [2,3]. DS can result in toxic biological effects and microbial drug resistance of different living organisms in water and even cause toxic effects on aquatic and terrestrial ecosystems, thereby posing a great threat to the environmental and human health [4,5].

The adsorption method is an effective means for removing organic pollutants from water [6]. This low-cost method is simple to operate and has no byproducts [7]. Common adsorption materials, such as bentonite, zeolite, and cellulose [8,9,10], possess advantages including a developed pore structure, high adsorption capacity, and low cost. However, issues such as poor adsorption regeneration and difficult solid–liquid separation greatly limit the practical application of these adsorbents. In recent years, magnetic nanoadsorbents have attracted extensive attention by compensating for the deficiencies of conventional adsorbents in water treatment [11]. Adsorbents containing magnetic nanoparticles can be easily separated with an external magnetic field [12,13].

Cellulose is the most abundant natural polysaccharide in nature and is advantageous for being cheap, biodegradable, and renewable [14,15,16,17]. Meanwhile, cellulose nanocrystals (CNC) can be extracted from cellulose and have a diameter of approximately 100 nm and a length ranging from hundreds of nanometers to several microns. The tensile strength of CNC was observed to be equivalent to that of cast iron. Moreover, CNC has been widely used an adsorbent for water treatment because of its high mechanical strength, adjustable surface chemical properties, and high specific surface area [18,19]. Because of the good hydrophilicity and stable colloidal properties of CNC, magnetic nanoparticles can be deposited on CNC by simple coprecipitation [20]. Therefore, magnetic adsorbents based on CNC have been widely studied [21,22]. These adsorbents are mostly beneficial in heavy metal removal, owing to the singleness of functional groups in magnetic nanoparticles and CNC [23]. Additionally, the adsorption mechanism involved in the removal of heavy metal ions by magnetic adsorbents is the electrostatic interaction between the positive and negative charges in the adsorbent and adsorbate [24]. However, the removal of organic pollutants in water, especially DS, using magnetic nanocrystalline cellulose (MCNC) has rarely been reported. This may be attributed to the lack of a functional group that can capture DS in MCNC. The adsorption capacity of chemically modified cellulose for various aquatic pollutants generally exceeds the adsorption capacity of unmodified cellulose. Many chemicals, such as inorganic nanoparticles, organic acids, and organic bases, have been used to modify cellulose.

Polyethyleneimine (PEI) is a functional compound that is widely used for adsorption and is popular for its high amino content and high reactivity. More importantly, past studies have shown that DS can be adsorbed by the rich ∓NH_2_ groups in PEI [25]. However, PEI requires a solid carrier, owing to its high water solubility. Therefore, PEI is often integrated into various solid adsorbents to improve their adsorption performance [26]. Lu et al. reported a type of aminated silica/polyvinyl alcohol/chitosan composite bead that was cross-linked with PEI, which exhibited remarkable DS removal ability [27]. The only functional group in CNC is a hydroxyl group, which cannot directly react with PEI. Therefore, a bridge that can react with both CNC and PEI is essential. [3-(2,3-epoxypropoxy) propyl] trimethoxysilane (EPPTMS) is a routine silane coupling agent containing silyloxy on one end, which can react with hydroxyl groups in cellulose via a dehydration–condensation reaction. The other end comprises the epoxy group, which can react with the amino groups in PEI [28].

In this study, we used EPPTMS to connect MCNC and PEI and to create an adsorbent that can effectively remove DS from water. The fabricated adsorbent was optimized based on the adsorption parameters, such as aqueous solution pH, adsorbent dosage, adsorption time, DS concentration, and adsorbent regeneration. The performance of our proposed adsorbent was also assessed and compared with previously reported adsorbents.

## 2. Materials and Methods

### 2.1. Materials

Polyethyleneimine (PEI, molecular weight, 600; purity, 99%), ferric chloride (FeCl_3_•6H_2_O, Purity 99%), ammonia water (NH_3_•H_2_O, content of NH_3_: 25–28%), ferrous chloride (FeCl_2_•4H_2_O, Purity 99%), diclofenac sodium (DS, Purity ≥ 99%), and [3-(2,3-epoxypropoxy) propyl] trimethoxysilane (EPPTMS, Purity ≥ 99%) were purchased from Aladdin (Shanghai, China). Cellulose nanocrystal (CNC, length 50–200 nm, diameter 5–20 nm, Crystallinity 88%) was purchased from Science K (Beijing, China). Deionized water was used as experimental water.

### 2.2. Methods

#### 2.2.1. Preparation of MCNC-PEI

MCNC was prepared using a coprecipitation method. Specifically, 2 g of CNC was uniformly and ultrasonically dispersed in deionized water for 5 min. Subsequently, an N2 atmosphere was introduced into the dispersion to remove the O2. Then, FeCl3 (4.8 g) and FeCl2 (1.8 g) were added to the dispersion, the temperature of the reaction system was maintained at 75 °C, and the pH was adjusted to 9 using ammonia. The pH value was measured using a pH test paper by dropping a small amount of reaction solution onto the test paper. The reaction proceeded for 30 min. The product was consecutively washed with water and ethanol and then freeze-dried [29].

MCNC-PEI was prepared as follows: EPPTMS was grafted onto the surface of MCNC via hydrolysis of the silanol groups and hydroxyl. Specifically, MCNC (1 g) and EPPTMS (0.75 g) were added to the 50 mL dimethylformamide (DMF) solution (containing 2 mL of water), and the mixture was stirred at 80 °C for 24 h under condensation reflux, which yielded MCNC-EPPTMS. Then, the reaction continued, following the addition of 1 g of PEI. After 24 h, the product was separated using a magnet and washed with DMF and ethanol. The adsorbent was finally freeze-dried for further study [30].

#### 2.2.2. Adsorption and Desorption Experiment

The DS solution was prepared from 5 to 500 mg/L concentrations, and the pH of the DS solution was adjusted by dilute acid and alkali solutions from 4.5 to 7.5. All adsorption experiments were done by mixing MCNC-PEI in a 20 mL DS solution. The dose of MCNC-PEI ranged from 5 mg to 30 mg. The adsorption temperature was then maintained at 25 °C. The adsorption properties at two other temperatures (20 °C and 30 °C) were investigated for isothermal model analysis. All adsorption experiments were also carried out in an oscillator with a disturbance frequency of 120 rpm.

Equations (1) and (2) were used to calculate the instantaneous adsorption capacity (q_t_) and equilibrium adsorption capacity (q_e_) of the adsorbent for DS [31].

(1) $$q_{t}=\frac{\left(C_{0}-C_{t}\right)V}{m}$$

(2) $$q_{e}=\frac{\left(C_{0}-C_{e}\right)V}{m}$$

where C_0_ (mg/L) is the initial concentration, C_t_ (mg/L) is the concentration of DS at time *t*, m (g) is the dose of MCNC-PEI, V (L) is the volume of the adsorbed solution, and C_e_ (mg/L) is the equilibrium concentration of DS.

The desorption experiments were conducted as follows: The DS that adsorbed on MCNC-PEI was eluted with NaOH (0.1 M). Then, MCNC-PEI-DS was added to the NaOH (0.1 M) solution, and ultrasonic vibrations were used to speed up the removal of DS from MCNC-PEI. The MCNC-PEI-DS was further eluted with an NaOH (0.1 M) solution several times until the DS could not be detected in the eluate. After freeze drying, the eluted MCNC-PEI was used to adsorb the DS under the same conditions. The operation was repeated four times, and the q_e_ of MCNC-PEI for DS was measured each time.

#### 2.2.3. Characterization

The physical structures of the adsorbents were analyzed using transmission electron microscopy (TEM, Hitachi SU8010, Tokyo, Japan). The samples observed by TEM were dispersed in ethanol and dropped onto a copper grid. Fourier transform infrared (FTIR, Burker TENSOR II, Karlsruhe, Germany) spectra were recorded to study the functional groups of the adsorbents. The samples were compressed with potassium bromide and analyzed by OMNIC™ Specta software. Furthermore, the surface compositions of MCNC-PEI before and after the adsorption of DS were determined by X-ray photoelectron spectroscopy (XPS, Thermo ESCALAB 250XI, Waltham, MA, USA) using monochromatic Al Ka radiation at 1486.6 eV. The results were analyzed by Casa XPS software. An X-ray diffractometer (XRD, Bruker D8 ADVANCE, Karlsruhe, Germany) was used to obtain the crystalline structure of the adsorbent. Lastly, the magnetic properties of the adsorbents were measured using a vibration sample magnetometer (VSM, Quantum Design PPMS DynaCool, Santiago, CA, USA) at a temperature of 25 °C and an external magnetic field of 20 kOe.

## 3. Results

### 3.1. Preparation Scheme of MCNC-PEI

**Figure 1:**
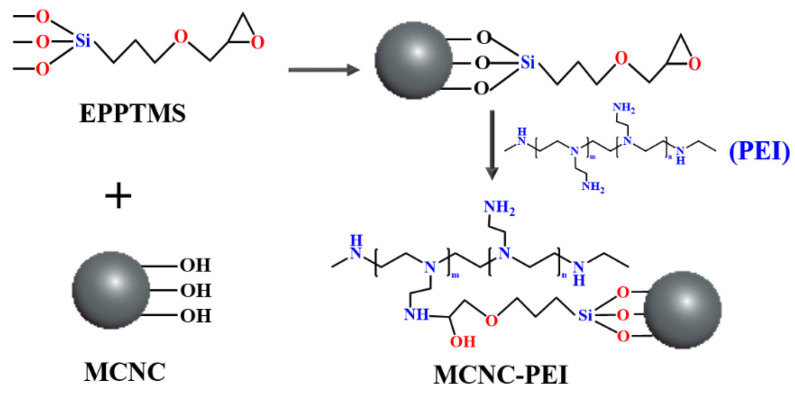
The fabrication scheme of MCNC-PEI.

In this study, MCNC was prepared by the coprecipitation method. Owing to the surface electronegativity of CNC, Fe^3+^ and Fe^2+^ can be enriched on the surface of CNC. After adjusting the pH to alkaline values, CNC loaded with magnetic nanoparticles was obtained. Magnetic adsorbents have unique advantages in terms of adsorbent separation. Moreover, the Si-OH bond formed by hydrolysis of the silane coupling agent can react with the hydroxyl group in MCNC by a dehydration–condensation reaction. Therefore, in this study, a silane coupling agent containing an epoxy group was grafted onto the MCNC. PEI is rich in amino groups, which can react with epoxy groups to graft PEI onto the MCNC. The reaction scheme of the preparation process of MCNC-PEI is shown in Figure 1.

### 3.2. Characterization

#### 3.2.1. TEM Analysis

**Figure 2:**
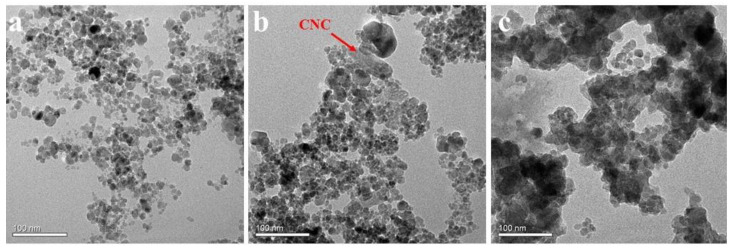
TEM of (**a**) Fe_3_O_4_, (**b**) MCNC, and (**c**) MCNC-PEI.

Figure 2a shows the TEM image of Fe_3_O_4_ nanoparticles. These magnetic nanoparticles are spherical and show significant aggregation. After the MCNC was prepared via the coprecipitation method, CNC with a larger particle size was attached to the Fe_3_O_4_ nanoparticles, as shown in Figure 2b. Meanwhile, Figure 2c shows the morphology of the MCNC-PEI. The MCNC was coated with a uniform cross-linked polymer network structure, which results from the PEI grafting on MCNC by the reaction between the PEI and epoxy group. In addition, the coverage of the polymer resulted to the aggregation of MCNC, so the particle size of MCNC-PEI increased.

#### 3.2.2. FTIR Analysis

**Figure 3:**
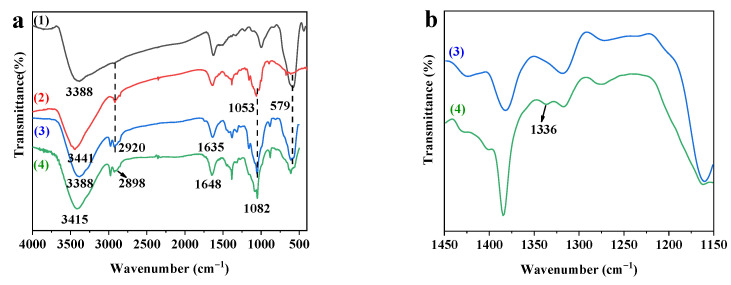
(**a**) FTIR spectra of (1) Fe_3_O_4_, (2) CNC, (3) MCNC, and (4) MCNC-PEI; (**b**) FTIR spectra of (3) MCNC and (4) MCNC-PEI at the wavenumber range of 1450–1150 cm^−1^.

FTIR spectra were used to further explain the changes in the functional groups of adsorbents. In the wavenumber range of 4000–400 cm^−1^, as shown in Figure 3a, the adsorption peak at 579 cm^−1^ is the characteristic absorption peak of Fe-O bands for stretching vibration in the crystalline lattice of Fe_3_O_4_. The characteristic absorption peak of –OH in Fe_3_O_4_ was at 3388 cm^−1^ [32]. The peak at 3441 cm^−1^ corresponds to the stretching vibration peak of the hydroxyl group in cellulose. Moreover, the peak at 2920 cm^−1^ corresponds to the stretching vibration of –CH. The peak at 1635 cm^−^^1^ corresponds to –OH bending vibration of absorbed water. The peak at 1053 cm^−^^1^ corresponds to the C–O–C pyranose ring stretching vibration [33,34]. The increase in the intensity of the band indicates increased cellulose content. These characteristic peaks of Fe_3_O_4_ and CNC are retained in the FTIR spectrum of MCNC, which confirms the successful preparation of MCNC. Figure 3b shows the FTIR spectrum of MCNC-PEI. In addition to the characteristic peaks of MCNC, the FTIR spectrum of MCNC-PEI also showed its characteristic absorption peaks. The peak at 1082 cm^−1^ corresponds to the stretching vibration of Si–O–Si [35], whereas the peak at 3415 cm^−^^1^ corresponds to the characteristic positions of –OH and –NH groups. The peak at 1648 cm^−^^1^ corresponds to the overlapping of the bending vibrations of N–H and –OH of absorbed water. The locally magnified image of Figure 3b at the wavenumber range of 1400–1150 cm^−^^1^. The small absorption peak at 1336 cm^−^^1^ corresponds to the stretching vibration of C–N due to the grafting of PEI [36]. The FTIR spectra further prove that MCNC-PEI was successfully prepared.

#### 3.2.3. XRD Analysis

**Figure 4:**
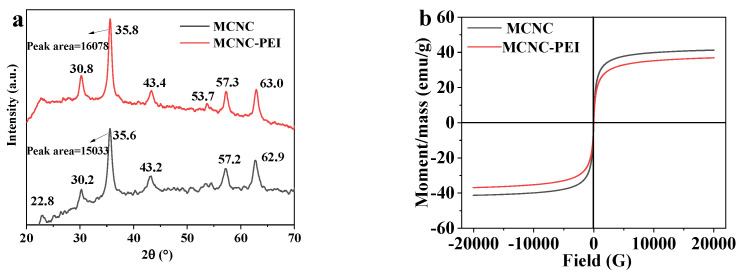
(**a**) XRD of MCNC and MCNC-PEI; (**b**) VSM of MCNC and MCNC-PEI.

XRD was used to reflect the changes in crystal structure within the composite adsorbent. As shown in the Figure 4a, the diffraction peak at 22.8° corresponds to the crystal plane (002) of CNC [37]. The six diffraction peaks at 30.2°, 35.6°, 43.2°, 53.7°, 57.2°, and 62.9° correspond to the crystal plane of Fe_3_O_4_ (220), (311), (400), (422), (511), and (440), respectively [38,39]. When MCNC was modified using the silane coupling agent and PEI, the diffraction peak at 62.7° increased to a larger angle, indicating that the particle size of MCNC was larger than that of MCNC-PEI, which is consistent with the conclusion of TEM analysis.

#### 3.2.4. VSM

The magnetic properties of the adsorbent are characterized by VSM. As shown in Figure 4b, the hysteresis loops of MCNC and MCNC-PEI exhibit the same change. Therefore, the modification process of MCNC by PEI does not influence the magnetic properties. Moreover, the saturation magnetization of MCNC is 41.21 emu/g, whereas the saturation magnetization of MCNC-PEI is 36.94 emu/g. The saturation magnetic strength of MCNC-PEI decreased, owing to the coating of PEI. In practice, MCNC-PEI can still be quickly separated from water.

#### 3.2.5. XPS Analysis

**Figure 5:**
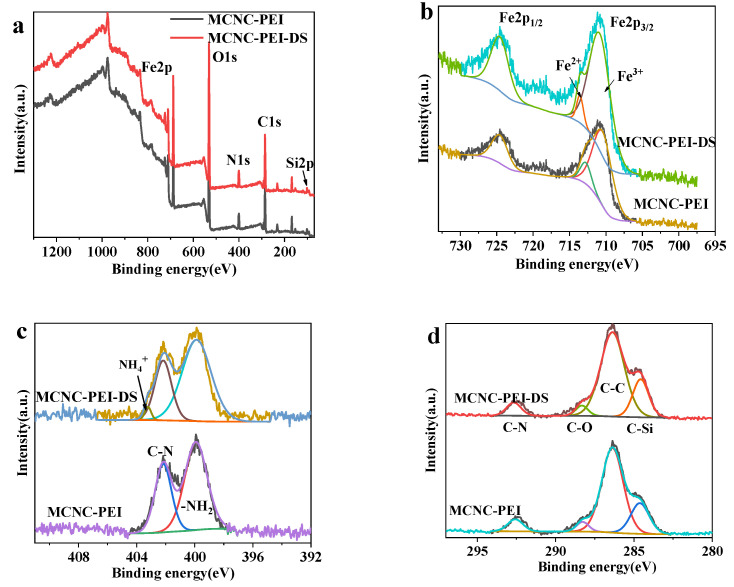
XPS spectrum of MCNC-PEI and MCNC-PEI loaded with DS. (**a**) Full spectrum; (**b**) Fe2p; (**c**) N1s; and (**d**) C1s.

The weak peak of Si 2p in Figure 5a was derived from the combination of the silane coupling agent and MCNC. Meanwhile, the Fe 2p_3/2_ peak can be fitted as two peaks at 710.5 eV and 712.8 eV, as shown in Figure 5b. These two peaks belong to Fe^3+^ and Fe^2+^ of Fe_3_O_4_, and the ratios of Fe^3+^/Fe^2+^ are 4.9 and 6.2, respectively. This ratio is slightly higher than the ratio of Fe^3+^/Fe^2+^ in Fe_3_O_4_, indicating that some of the magnetic nanoparticles in MCNC-PEI are oxidized, and the particles are further oxidized in the adsorption process. Figure 5c shows the XPS of N 1s. This element originated from PEI, which further confirms that PEI is successfully coated on MCNC. After adsorbing DS, a part of –NH_2_ is converted to –NH_3_^+^. This indicates that the combination of PEI and DS is in the form of –NH_3_^+^. Figure 5d shows the C 1s peak. The area ratio of the C–C peak increased slightly, while the other ratios decreased. This is because DS is loaded on MCNC-PEI, which changes the area ratio of each fitting peak of C 1s. The XPS results illustrate that PEI successfully modified the MCNC and plays an important role in DS removal.

### 3.3. Optimization of Adsorption Conditions

**Figure 6:**
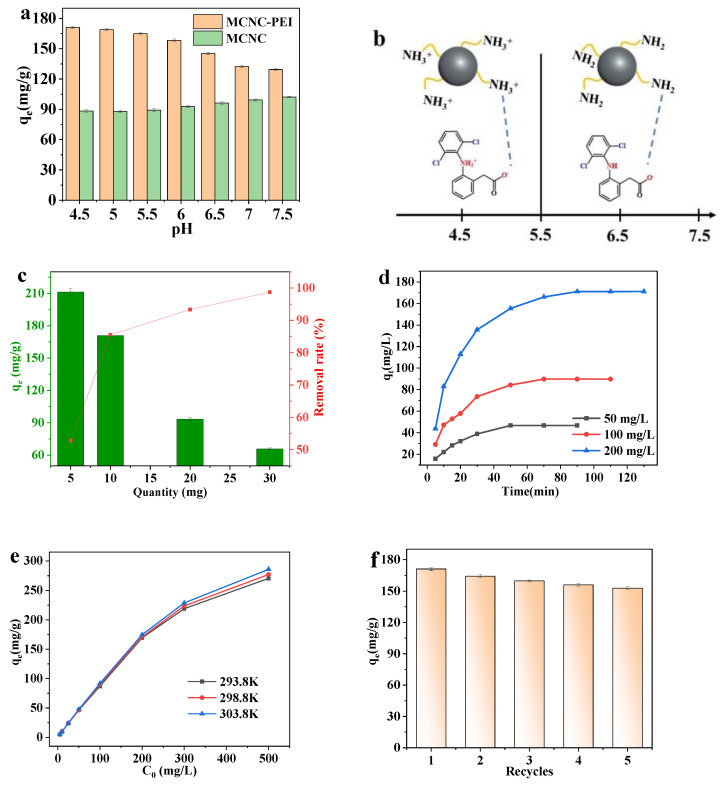
(**a**) Effect of PH on the adsorption capacity of MCNC and MCNC-PEI for DS; (**b**) schematic of the adsorption mechanism of MCNC-PEI for DS; effect of (**c**) adsorbent dosage, (**d**) adsorption time, and (**e**) initial concentration of DS on adsorption capacity; and (**f**) effect of recycling time on the adsorption capacity of MCNC-PEI for DS.

Solution pH is one of the important factors affecting adsorption performance. To further study the adsorption mechanism, MCNC and MCNC-PEI were placed in DS solutions with different pH values. The DS adsorption capacities of MCNC and MCNC-PEI at different pH values showed opposite trends. Hydrogen bonds can be formed between MCNC-PEI and PEI. In a more acidic condition, increasing free hydrogen ions can destroy the hydrogen bonds, and excessive hydroxyl radicals in an alkaline solution can result in the disassociation of hydrogen bonds. Therefore, the adsorption capacity of MCNC for DS was higher under neutral conditions. As shown in Figure 6a, the removal ability of DS by MCNC-PEI was more optimized in a slightly acidic environment (pH = 4.5–5.5), where the carboxyl group in DS is dissociated, whereas the amino group in PEI is in a quaternary ammonium state. Therefore, MCNC-PEI can easily capture DS through charge action. Figure 6b shows the states of MCNC-PEI and DS at different pH values [40].

Furthermore, the adsorption capacity was significantly affected by the quantity of adsorbent. As shown in Figure 6c, a higher adsorption capacity (211.4 mg/g) was obtained with a lower adsorbent dose (0.5 mg/L), whereas the removal rate of DS is only 52.85%. Increasing the adsorbent dose also gradually increased the DS removal rate. However, it was accompanied by a decrease in adsorption capacity. Therefore, when selecting the adsorbent dosage, the DS concentration must be considered to ensure optimal removal efficiency. Based on the experimental results, we chose the amount of adsorbent as 1 mg/L for further tests.

The time taken for adsorption to reach equilibrium was closely linked to the concentration of DS. The adsorption capacity of DS at different adsorption times was studied, with concentrations of DS ranging from 50 to 200 mg/L; the results are shown in Figure 6d. As the DS concentration increased, the time required for adsorption to reach equilibrium also increased. When the concentration was 50 mg/L, it took 30 min for adsorption to reach equilibrium and up to 90 min at a DS concentration of 200 mg/L. In addition, a higher concentration of DS caused a greater adsorption capacity of MCNC-PEI for DS. The adsorption process is the capture process of DS by MCNC-PEI; therefore, a high DS concentration prolongs the capture process.

Moreover, the adsorption capacities of MCNC-PEI at different temperatures and DS concentrations are shown in Figure 6e. The adsorption rate gradually decreased as the DS concentration increased. This indicates that the adsorption capacity of the adsorbent tends to be saturated. In addition, appropriately increasing the temperature increases the adsorption capacity of MCNC-PEI because the temperature increase accelerates the molecular motion, thus increasing the binding probability between DS and the adsorbent, which leads to a higher adsorption capacity.

Adsorbent regeneration pertains to the use of physical or chemical methods to separate or decompose the adsorbate on the adsorbent surface and to restore the adsorption performance and reusability. A regenerating adsorbent reduces the treatment cost. In this study, the regeneration of MCNC-PEI-DS was achieved by alkali extraction. The adsorption capacity of MCNC-PEI for DS declined by 9.9% after it had been used five times, as shown in Figure 6f. This shows that the adsorption capacity of MCNC-PEI can maintain long-term stability.

According to Table 1, MCNC-PEI has a higher adsorption capacity for DS compared to previously reported adsorbents. This is related to a variety of adsorption mechanisms between MCNC-PEI and DS, including hydrogen bonds and electrostatic interactions. In addition, as a type of polyamine polymer, PEI has a high DS binding ability, and its advantage is significantly higher than that of chitosan.

### 3.4. Adsorption Mechanism

Adsorption is a process whereby adsorbates are enriched with solid adsorbents, either by physical or chemical means. Physical adsorption mainly depends on van der Waals forces and does not involve electron transfer. This adsorption mode has a low capacity, and the adsorption and desorption processes are rapid. Meanwhile, chemical adsorption involves the complexation of adsorbate molecules with functional groups on the surface of the adsorbent [44]. This process requires activation energy. Compared with physical desorption, the adsorption and separation rates are slower. In addition, the adsorption rate increases as the temperature increases [45,46].

**Figure 7:**
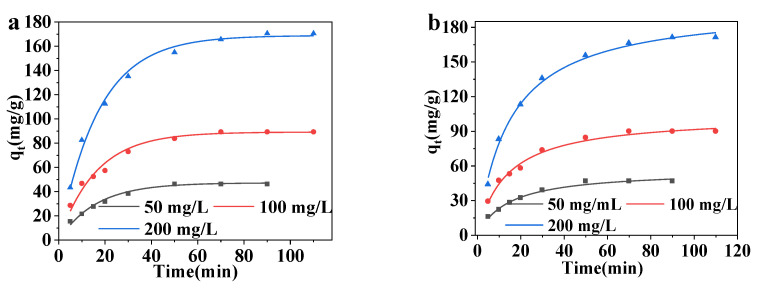
The fitting results of MCNC-PEI adsorbing DS by (**a**) pseudo-first-order and (**b**) pseudo-second-order isotherm models.

The kinetic model explores and predicts the adsorption mechanism. The data for MCNC-PEI adsorption of DS was fitted by the pseudo-first-order and pseudo-second-order kinetic models. The pseudo-first-order model describes an adsorption process that is controlled by diffusion and involves only a single adsorption site. The pseudo-second-order kinetic model accounts for the adsorption process that involves the sharing or transfer of electron pairs between the adsorbate and adsorbent, and there are two binding sites on the adsorbent surface [47]. The equations for the two adsorption kinetic models are as follows [48]:

(3) $$q_{t}=q_{e}\left(1-e^{-k_{1}t}\right)$$

(4) $$q_{t}=\frac{q_{e}^{2}k_{2}t}{1+{\mathrm{tk}}_{2}q_{e}}$$

where k_1_ and k_2_ are the respective adsorption rate constants of the two adsorption kinetic models. The results are shown in Figure 7, and the relevant parameters are presented in Table 2. Parametric analysis of these models show that the adsorption process of MCNC-PEI for DS is more consistent with the pseudo-second-order kinetic model; therefore, the adsorption process involves the sharing or transfer of electron pairs.

The adsorption isotherm model includes the Freundlich and Langmuir isotherm models. The Freundlich isotherm model is used to describe the mechanism of multilayer adsorption and heterogeneous adsorption systems, so it has a wide range of applications in the study of adsorption mechanisms. Meanwhile, the Langmuir adsorption isotherm model is used to describe monolayer adsorption. The basic assumptions of the model are as follows: the adsorbent surface energy is uniform, the intermolecular force of the adsorbate is disregarded, and the adsorption process is reversible [49]. The adsorption capacities for the Freundlich and Langmuir isotherm models are given by Equations (5) and (6), respectively [50]:

(5) $$q_{e}=K_{F}C_{e}^{n}$$

(6) $$q_{e}=\frac{q_{m}K_{L}C_{e}}{1+K_{L}C_{e}}$$

where q_m_ (mg/g) represents the maximum adsorption capacity of MCNC-PEI, K_L_ (L/mg) is a constant for the adsorption affinity; and n and K_F_ (mg/g) are the adsorption strength and constant, respectively.

**Figure 8:**
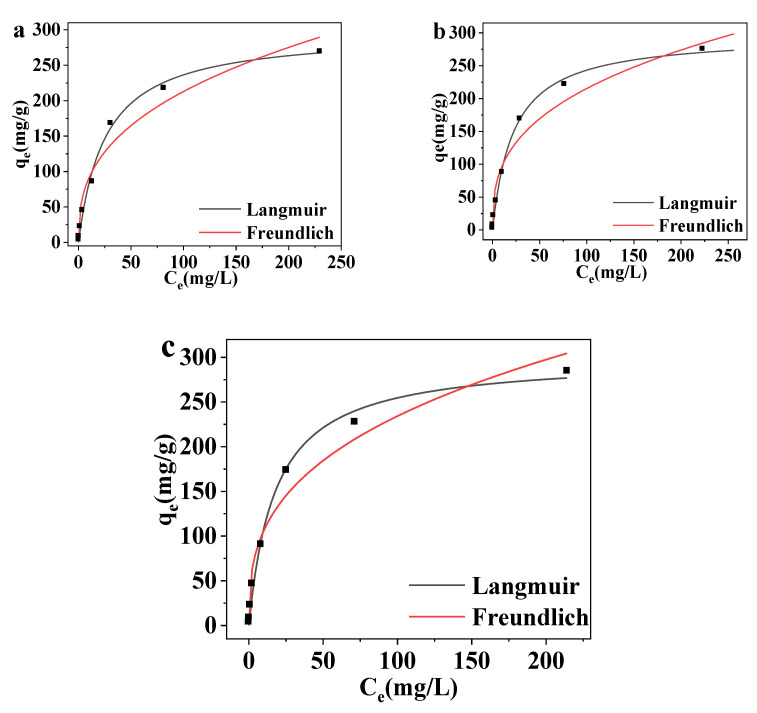
Adsorption isotherm model-fitting curves for MCNC-PEI adsorbing Cu(II) at (**a**) 293.8K, (**b**) 293.8K, and (**c**) 293.8K.

These isothermal adsorption models were used to fit the data for DS adsorption by MCNC-PEI. The fitting curves of the adsorption data of MCNC-PEI at different temperatures are shown in Figure 8, and the relevant parameters are listed in Table 3. The nonlinear fitting coefficients (R^2^) indicate that the adsorption process is consistent with the Langmuir isotherm model. Moreover, the DS adsorption process of MCNC-PEI is monolayer adsorption, and the active sites on the adsorbent surface were uniform. The q_max_ fitted by the Langmuir isotherm equation at 293.2 K is 297.49 mg/g, and the value increases as the adsorption temperature increases, which shows that the adsorption process is endothermic. Increasing the temperature contributes to improved adsorption performance of MCNC-PEI on DS. The DS removal advantage of MCNC-PEI was assessed in comparison with adsorbents that were previously reported in literature. The comparison was based on the q_max_ value obtained from the Langmuir isotherm model, and the values are presented in Table 4. The proposed MCNC-PEI clearly has the highest maximum absorption capacity for DS among other absorbents in the literature.

## 4. Conclusions

An environmentally friendly and convenient adsorbent was constructed in this work. MCNC-PEI shows good paramagnetism and can be quickly separated and recovered from liquid by an external magnetic field. The adsorption process of MCNC-PEI on DS relies mainly on hydrogen bonding and charge interaction. Experimental results confirm that CNC has a strong DS removal capacity. The optimal amount of adsorbent to use is 1 mg/mL. The adsorption process of MCNC-PEI on DS is consistent with the pseudo-second-order kinetic and Langmuir isotherm models. The maximum adsorption capacity can reach 300.19 mg/g at 30 °C. In addition, MCNC-PEI has shown excellent recyclability. Adsorbents are playing a considerable role in the treatment of water environments the efficient and fast composite adsorbents gradually show their advantages. Based on these advantages, MCNC-PEI is a promising adsorbent material for the removal of DS.

## Figures and Tables

**Table 1:** Comparison of the diclofenac adsorption capacity of MCNC-PEI with that of adsorbents reported in the literature, as well as possible interaction mechanisms.

Sample	Experimental Conditions		q_m_(mg/g)	Main Interaction Mechanisms	Reference
	pH	Temperature (°C)			
ZnFe_2_O_4_/chitosan	4.0	25	10.1	Hydrogen bonds and electrostatic interactions	[41]
CS-MEDSP	4.0	40	120.0	Electrostatic interactions	[42]
Functionalized silica with ionic liquid	6.7	25	273.8	Covalent bonds	[43]
MCNC-PEI	4.5	25	299.93	Hydrogen bonds and electrostatic interactions	This work

**Table 2:** Adsorption kinetic model parameters fitted for the adsorption process of MCNC-PEI for DS.

DS Concentration	Pseudo-First-Order			Pseudo-Second-Order		
	Δq (mg/g)	k_1_ (min^−1^)	R^2^	Δq (mg/g)	K_2_ (g mg^−1^min^−1^)	R^2^
50 mg/L	−0.73	0.075	0.972	6.73	1.72 × 10^−3^	0.993
100 mg/L	−1.04	0.066	0.976	12.75	7.93 × 10^−4^	0.992
200 mg/L	−2.67	0.058	0.991	23.98	3.57 × 10^−4^	0.993

**Table 3:** Fitting parameters of the adsorption isotherm model examined for the adsorption process of MCNC-PEI for DS.

Temperature	Langmuir			Freundlich		
	q_m_ (mg/g)	K_L_ (min^−1^)	R^2^	K_F_ (mg/g)	n (g mg^−1^min^−1^)	R^2^
293.8 K	297.49	0.039	0.991	38.79	2.70	0.956
298.8 K	299.93	0.044	0.995	41.49	2.75	0.933
303.8 K	300.19	0.056	0.991	47.90	2.90	0.964

**Table 4:** Adsorption capacities of reported adsorbents for DS compared to MCNC-PEI.

Adsorbents	q_max_ (mg/g)	Reference
g-C_3_N_4_/MoO_3_ activated carbon (SAC) CNCs60 Fe_3_O_4_@SiO_2_/SiHTCC MCNC-PEI	162 178.89 107.87 240.4 299.93	[51] [52] [53] [54] This work

## Data Availability

Not applicable.
